# Supplementary material for: The Current Concept of Paternal Bonding: A Systematic Scoping Review
Source: Healthcare (Basel). 2022 Nov 11;10(11):2265. doi: 10.3390/healthcare10112265 (PMC9690989; doi:10.3390/healthcare10112265)
Supplement: Supplementary file 1 [file healthcare-10-02265-s001.zip › healthcare-1997280-supplementary.pdf]

**Supplementary: Search strategies**

*I.* ***PubMed***

("paternal bonding"[tw] OR "paternal bond"[tw] OR "paternal bonds"[tw] OR "paternal bond*"[tw] OR "father bond*"[tw] OR "father infant bond*"[tw] OR "father daughter bond*"[tw] OR "father son bond*"[tw] OR "father to infant bond*"[tw] OR "father to daughter bond*"[tw] OR "father to son bond*"[tw] OR "paternal infant bond*"[tw] OR "paternal daughter bond*"[tw] OR "paternal son bond*"[tw] OR "paternal to infant bond*"[tw] OR "paternal to daughter bond*"[tw] OR "paternal to son bond*"[tw] OR "paternal fetus bond*"[tw] OR "father fetus bond*"[tw] OR "paternal foetus bond*"[tw] OR "father foetus bond*"[tw] OR "paternal to fetus bond*"[tw] OR "father to fetus bond*"[tw] OR "paternal to foetus bond*"[tw] OR "father to foetus bond*"[tw] OR "paternal child bond*"[tw] OR "father child bond*"[tw] OR "paternal to child bond*"[tw] OR "father to child bond*"[tw] OR "paternal attach*"[tw] OR "father attach*"[tw] OR "father infant attach*"[tw] OR "father daughter attach*"[tw] OR "father son attach*"[tw] OR "father to infant attach*"[tw] OR "father to daughter attach*"[tw] OR "father to son attach*"[tw] OR "paternal infant attach*"[tw] OR "paternal daughter attach*"[tw] OR "paternal son attach*"[tw] OR "paternal to infant attach*"[tw] OR "paternal to daughter attach*"[tw] OR "paternal to son attach*"[tw] OR "paternal fetus attach*"[tw] OR "father fetus attach*"[tw] OR "paternal foetus attach*"[tw] OR "father foetus attach*"[tw] OR "paternal to fetus attach*"[tw] OR "father to fetus attach*"[tw] OR "paternal to foetus attach*"[tw] OR "father to foetus attach*"[tw] OR "paternal child attach*"[tw] OR "father child attach*"[tw] OR "paternal to child attach*"[tw] OR "father to child attach*"[tw] OR "paternal adolescent bond*"[tw] OR "father adolescent bond*"[tw] OR "paternal to adolescent bond*"[tw] OR

"father to adolescent bond*"[tw] OR "paternal adolescent attach*"[tw] OR "father adolescent attach*"[tw] OR "paternal to adolescent attach*"[tw] OR "father to adolescent attach*"[tw] OR ("Father-Child Relations"[Mesh] AND "Object Attachment"[Mesh]) OR (("bonding"[ti] OR "bond"[ti] OR "bonds"[ti] OR "bonded"[ti] OR bond*[ti]) AND (father*[ti] OR paternal*[ti])) OR (("attachment"[ti] OR "attach*"[ti]) AND (father*[ti] OR paternal*[ti])) OR (("bonding"[tw] OR "bond"[tw] OR "bonds"[tw] OR "bonded"[tw] OR bond*[tw] OR attach*[tw]) AND ("infant*"[tw] OR "child*"[tw] OR "daughter*"[tw] OR "son"[tw] OR "sons"[tw] OR "fetus*"[tw] OR "foetus*"[tw] OR "adolescent*"[tw]) AND (father*[ti] OR paternal*[ti])) OR (("bonding"[ti] OR "bond"[ti] OR "bonds"[ti] OR "bonded"[ti] OR bond*[ti] OR attach*[ti]) AND ("infant*"[tw] OR "child*"[tw] OR "daughter*"[tw] OR "son"[tw] OR "sons"[tw] OR "fetus*"[tw] OR "foetus*"[tw] OR "adolescent*"[tw]) AND (father*[tw] OR paternal*[tw])))

### *II. MEDLINE via OVID*

("paternal bonding".mp OR "paternal bond".mp OR "paternal bonds".mp OR "paternal bond*".mp OR "father bond*".mp OR "father infant bond*".mp OR "father daughter bond*".mp OR "father son bond*".mp OR "father to infant bond*".mp OR "father to daughter bond*".mp OR "father to son bond*".mp OR "paternal infant bond*".mp OR "paternal daughter bond*".mp OR "paternal son bond*".mp OR "paternal to infant bond*".mp OR "paternal to daughter bond*".mp OR "paternal to son bond*".mp OR "paternal fetus bond*".mp OR "father fetus bond*".mp OR "paternal foetus bond*".mp OR "father foetus bond*".mp OR "paternal to fetus bond*".mp OR "father to fetus bond*".mp OR "paternal to foetus bond*".mp OR "father to foetus bond*".mp OR "paternal child bond*".mp OR "father child bond*".mp OR "paternal to child bond*".mp OR "father to child bond*".mp OR "paternal attach*".mp OR "father attach*".mp OR "father infant attach*".mp OR "father

daughter attach*".mp OR "father son attach*".mp OR "father to infant attach*".mp OR "father to daughter attach*".mp OR "father to son attach*".mp OR "paternal infant attach*".mp OR "paternal daughter attach*".mp OR "paternal son attach*".mp OR "paternal to infant attach*".mp OR "paternal to daughter attach*".mp OR "paternal to son attach*".mp OR "paternal fetus attach*".mp OR "father fetus attach*".mp OR "paternal foetus attach*".mp OR "father foetus attach*".mp OR "paternal to fetus attach*".mp OR "father to fetus attach*".mp OR "paternal to foetus attach*".mp OR "father to foetus attach*".mp OR "paternal child attach*".mp OR "father child attach*".mp OR "paternal to child attach*".mp OR "father to child attach*".mp OR "paternal adolescent bond*".mp OR "father adolescent bond*".mp OR "paternal to adolescent bond*".mp OR "father to adolescent bond*".mp OR "paternal adolescent attach*".mp OR "father adolescent attach*".mp OR "paternal to adolescent attach*".mp OR "father to adolescent attach*".mp OR ("Father-Child Relations"/ AND "Object Attachment"/) OR (("bonding".ti OR "bond".ti OR "bonds".ti OR "bonded".ti OR bond*.ti) AND (father*.ti OR paternal*.ti)) OR (("attachment".ti OR "attach*".ti) AND (father*.ti OR paternal*.ti)) OR (("bonding".mp OR "bond".mp OR "bonds".mp OR "bonded".mp OR bond*.mp OR attach*.mp) AND ("infant*".mp OR "child*".mp OR "daughter*".mp OR "son".mp OR "sons".mp OR "fetus*".mp OR "foetus*".mp OR "adolescent*".mp) AND (father*.ti OR paternal*.ti)) OR (("bonding".ti OR "bond".ti OR "bonds".ti OR "bonded".ti OR bond*.ti OR attach*.ti) AND ("infant*".mp OR "child*".mp OR "daughter*".mp OR "son".mp OR "sons".mp OR "fetus*".mp OR "foetus*".mp OR "adolescent*".mp) AND (father*.mp OR paternal*.mp)) OR (("bonding".mp OR "bond".mp OR "bonds".mp OR "bonded".mp OR bond*.mp) ADJ5 (father*.mp OR paternal*.mp)))

### *III. Embase*

("paternal bonding".mp OR "paternal bond".mp OR "paternal bonds".mp OR "paternal bond*".mp OR "father bond*".mp OR "father infant bond*".mp OR "father daughter bond*".mp OR "father son bond*".mp OR "father to infant bond*".mp OR "father to daughter bond*".mp OR "father to son bond*".mp OR "paternal infant bond*".mp OR "paternal daughter bond*".mp OR "paternal son bond*".mp OR "paternal to infant bond*".mp OR "paternal to daughter bond*".mp OR "paternal to son bond*".mp OR "paternal fetus bond*".mp OR "father fetus bond*".mp OR "paternal foetus bond*".mp OR "father foetus bond*".mp OR "paternal to fetus bond*".mp OR "father to fetus bond*".mp OR "paternal to foetus bond*".mp OR "father to foetus bond*".mp OR "paternal child bond*".mp OR "father child bond*".mp OR "paternal to child bond*".mp OR "father to child bond*".mp OR "paternal attach*".mp OR "father attach*".mp OR "father infant attach*".mp OR "father daughter attach*".mp OR "father son attach*".mp OR "father to infant attach*".mp OR "father to daughter attach*".mp OR "father to son attach*".mp OR "paternal infant attach*".mp OR "paternal daughter attach*".mp OR "paternal son attach*".mp OR "paternal to infant attach*".mp OR "paternal to daughter attach*".mp OR "paternal to son attach*".mp OR "paternal fetus attach*".mp OR "father fetus attach*".mp OR "paternal foetus attach*".mp OR "father foetus attach*".mp OR "paternal to fetus attach*".mp OR "father to fetus attach*".mp OR "paternal to foetus attach*".mp OR "father to foetus attach*".mp OR "paternal child attach*".mp OR "father child attach*".mp OR "paternal to child attach*".mp OR "father to child attach*".mp OR "paternal adolescent bond*".mp OR "father adolescent bond*".mp OR "paternal to adolescent bond*".mp OR "father to adolescent bond*".mp OR "paternal adolescent attach*".mp OR "father adolescent attach*".mp OR "paternal to adolescent attach*".mp OR "father to adolescent attach*".mp OR ("father child relation"/ AND exp "Social Bonding"/) OR (("bonding".ti OR "bond".ti OR "bonds".ti OR "bonded".ti OR bond*.ti) AND (father*.ti OR paternal*.ti)) OR (("attachment".ti OR "attach*".ti) AND

(father*.ti OR paternal*.ti)) OR (("bonding".mp OR "bond".mp OR "bonds".mp OR "bonded".mp OR bond*.mp OR attach*.mp) AND ("infant*".mp OR "child*".mp OR "daughter*".mp OR "son".mp OR "sons".mp OR "fetus*".mp OR "foetus*".mp OR "adolescent*".mp) AND (father*.ti OR paternal*.ti)) OR (("bonding".ti OR "bond".ti OR "bonds".ti OR "bonded".ti OR bond*.ti OR attach*.ti) AND ("infant*".mp OR "child*".mp OR "daughter*".mp OR "son".mp OR "sons".mp OR "fetus*".mp OR "foetus*".mp OR "adolescent*".mp) AND (father*.mp OR paternal*.mp)) OR (("bonding".mp OR "bond".mp OR "bonds".mp OR "bonded".mp OR bond*.mp) ADJ5 (father*.mp OR paternal*.mp))) NOT (conference review or conference abstract).pt

*IV.* ***Web of Science***

(ts=("paternal bonding" OR "paternal bond" OR "paternal bonds" OR "paternal bond*" OR "father bond*" OR "father infant bond*" OR "father daughter bond*" OR "father son bond*" OR "father to infant bond*" OR "father to daughter bond*" OR "father to son bond*" OR "paternal infant bond*" OR "paternal daughter bond*" OR "paternal son bond*" OR "paternal to infant bond*" OR "paternal to daughter bond*" OR "paternal to son bond*" OR "paternal fetus bond*" OR "father fetus bond*" OR "paternal foetus bond*" OR "father foetus bond*" OR "paternal to fetus bond*" OR "father to fetus bond*" OR "paternal to foetus bond*" OR "father to foetus bond*" OR "paternal child bond*" OR "father child bond*" OR "paternal to child bond*" OR "father to child bond*" OR "paternal attach*" OR "father attach*" OR "father infant attach*" OR "father daughter attach*" OR "father son attach*" OR "father to infant attach*" OR "father to daughter attach*" OR "father to son attach*" OR "paternal infant attach*" OR "paternal daughter attach*" OR "paternal son attach*" OR "paternal to infant attach*" OR "paternal to daughter attach*" OR "paternal to son attach*" OR "paternal fetus attach*" OR "father fetus attach*" OR "paternal foetus attach*" OR "father foetus attach*"

OR "paternal to fetus attach*" OR "father to fetus attach*" OR "paternal to foetus attach*" OR "father to foetus attach*" OR "paternal child attach*" OR "father child attach*" OR "paternal to child attach*" OR "father to child attach*" OR "paternal adolescent bond*" OR "father adolescent bond*" OR "paternal to adolescent bond*" OR "father to adolescent bond*" OR "paternal adolescent attach*" OR "father adolescent attach*" OR "paternal to adolescent attach*" OR "father to adolescent attach*") OR (ti=("bonding" OR "bond" OR "bonds" OR "bonded" OR bond*) AND ti=(father* OR paternal*)) OR (ti=("attachment" OR "attach*") AND ti=(father* OR paternal*)) OR (ts=("bonding" OR "bond" OR "bonds" OR "bonded" OR bond* OR attach*) AND ts=("infant*" OR "child*" OR "daughter*" OR "son" OR "sons" OR "fetus*" OR "foetus*" OR "adolescent*") AND ti=(father* OR paternal*)) OR (ti=("bonding" OR "bond" OR "bonds" OR "bonded" OR bond* OR attach*) AND ts=("infant*" OR "child*" OR "daughter*" OR "son" OR "sons" OR "fetus*" OR "foetus*" OR "adolescent*") AND ts=(father* OR paternal*)) OR ts=(("bonding" OR "bond" OR "bonds" OR "bonded" OR bond*) NEAR/2 (father* OR paternal*))) NOT dt=(conference abstract)

*V.* ***Cochrane***

(("paternal bonding" OR "paternal bond" OR "paternal bonds" OR "paternal bond*" OR "father bond*" OR "father infant bond*" OR "father daughter bond*" OR "father son bond*" OR "father to infant bond*" OR "father to daughter bond*" OR "father to son bond*" OR "paternal infant bond*" OR "paternal daughter bond*" OR "paternal son bond*" OR "paternal to infant bond*" OR "paternal to daughter bond*" OR "paternal to son bond*" OR "paternal fetus bond*" OR "father fetus bond*" OR "paternal foetus bond*" OR "father foetus bond*" OR "paternal to fetus bond*" OR "father to fetus bond*" OR "paternal to foetus bond*" OR "father to foetus bond*" OR "paternal child bond*" OR "father child bond*" OR "paternal to

child bond*" OR "father to child bond*" OR "paternal attach*" OR "father attach*" OR "father infant attach*" OR "father daughter attach*" OR "father son attach*" OR "father to infant attach*" OR "father to daughter attach*" OR "father to son attach*" OR "paternal infant attach*" OR "paternal daughter attach*" OR "paternal son attach*" OR "paternal to infant attach*" OR "paternal to daughter attach*" OR "paternal to son attach*" OR "paternal fetus attach*" OR "father fetus attach*" OR "paternal foetus attach*" OR "father foetus attach*" OR "paternal to fetus attach*" OR "father to fetus attach*" OR "paternal to foetus attach*" OR "father to foetus attach*" OR "paternal child attach*" OR "father child attach*" OR "paternal to child attach*" OR "father to child attach*" OR "paternal adolescent bond*" OR "father adolescent bond*" OR "paternal to adolescent bond*" OR "father to adolescent bond*" OR "paternal adolescent attach*" OR "father adolescent attach*" OR "paternal to adolescent attach*" OR "father to adolescent attach*"):ti,ab,kw OR (("bonding" OR "bond" OR "bonds" OR "bonded" OR bond*) AND (father* OR paternal*)):ti OR (("attachment" OR "attach*") AND (father* OR paternal*)):ti OR (("bonding" OR "bond" OR "bonds" OR "bonded" OR bond* OR attach*):ti,ab,kw AND ("infant*" OR "child*" OR "daughter*" OR "son" OR "sons" OR "fetus*" OR "foetus*" OR "adolescent*"):ti,ab,kw AND (father* OR paternal*):ti) OR (("bonding" OR "bond" OR "bonds" OR "bonded" OR bond* OR attach*):ti AND ("infant*" OR "child*" OR "daughter*" OR "son" OR "sons" OR "fetus*" OR "foetus*" OR "adolescent*"):ti,ab,kw AND (father* OR paternal*):ti,ab,kw) OR (("bonding" OR "bond" OR "bonds" OR "bonded" OR bond*) NEAR/2 (father* OR paternal*)):ti,ab,kw) NOT (conference abstract):pt

### *VI.* ***Emcare***

("paternal bonding".mp OR "paternal bond".mp OR "paternal bonds".mp OR "paternal bond*".mp OR "father bond*".mp OR "father infant bond*".mp OR "father daughter

bond*".mp OR "father son bond*".mp OR "father to infant bond*".mp OR "father to daughter bond*".mp OR "father to son bond*".mp OR "paternal infant bond*".mp OR "paternal daughter bond*".mp OR "paternal son bond*".mp OR "paternal to infant bond*".mp OR "paternal to daughter bond*".mp OR "paternal to son bond*".mp OR "paternal fetus bond*".mp OR "father fetus bond*".mp OR "paternal foetus bond*".mp OR "father foetus bond*".mp OR "paternal to fetus bond*".mp OR "father to fetus bond*".mp OR "paternal to foetus bond*".mp OR "father to foetus bond*".mp OR "paternal child bond*".mp OR "father child bond*".mp OR "paternal to child bond*".mp OR "father to child bond*".mp OR "paternal attach*".mp OR "father attach*".mp OR "father infant attach*".mp OR "father daughter attach*".mp OR "father son attach*".mp OR "father to infant attach*".mp OR "father to daughter attach*".mp OR "father to son attach*".mp OR "paternal infant attach*".mp OR "paternal daughter attach*".mp OR "paternal son attach*".mp OR "paternal to infant attach*".mp OR "paternal to daughter attach*".mp OR "paternal to son attach*".mp OR "paternal fetus attach*".mp OR "father fetus attach*".mp OR "paternal foetus attach*".mp OR "father foetus attach*".mp OR "paternal to fetus attach*".mp OR "father to fetus attach*".mp OR "paternal to foetus attach*".mp OR "father to foetus attach*".mp OR "paternal child attach*".mp OR "father child attach*".mp OR "paternal to child attach*".mp OR "father to child attach*".mp OR "paternal adolescent bond*".mp OR "father adolescent bond*".mp OR "paternal to adolescent bond*".mp OR "father to adolescent bond*".mp OR "paternal adolescent attach*".mp OR "father adolescent attach*".mp OR "paternal to adolescent attach*".mp OR "father to adolescent attach*".mp OR ("father child relation"/ AND exp "Social Bonding"/) OR (("bonding".ti OR "bond".ti OR "bonds".ti OR "bonded".ti OR bond*.ti) AND (father*.ti OR paternal*.ti)) OR (("attachment".ti OR "attach*".ti) AND (father*.ti OR paternal*.ti)) OR (("bonding".mp OR "bond".mp OR "bonds".mp OR "bonded".mp OR bond*.mp OR attach*.mp) AND ("infant*".mp OR "child*".mp OR

"daughter*".mp OR "son".mp OR "sons".mp OR "fetus*".mp OR "foetus*".mp OR "adolescent*".mp) AND (father*.ti OR paternal*.ti)) OR (("bonding".ti OR "bond".ti OR "bonds".ti OR "bonded".ti OR bond*.ti OR attach*.ti) AND ("infant*".mp OR "child*".mp OR "daughter*".mp OR "son".mp OR "sons".mp OR "fetus*".mp OR "foetus*".mp OR "adolescent*".mp) AND (father*.mp OR paternal*.mp)) OR (("bonding".mp OR "bond".mp OR "bonds".mp OR "bonded".mp OR bond*.mp) ADJ5 (father*.mp OR paternal*.mp)))

*VII.* ***PsycINFO***

(TX("paternal bonding" OR "paternal bond" OR "paternal bonds" OR "paternal bond*" OR "father bond*" OR "father infant bond*" OR "father daughter bond*" OR "father son bond*" OR "father to infant bond*" OR "father to daughter bond*" OR "father to son bond*" OR "paternal infant bond*" OR "paternal daughter bond*" OR "paternal son bond*" OR "paternal to infant bond*" OR "paternal to daughter bond*" OR "paternal to son bond*" OR "paternal fetus bond*" OR "father fetus bond*" OR "paternal foetus bond*" OR "father foetus bond*" OR "paternal to fetus bond*" OR "father to fetus bond*" OR "paternal to foetus bond*" OR "father to foetus bond*" OR "paternal child bond*" OR "father child bond*" OR "paternal to child bond*" OR "father to child bond*" OR "paternal attach*" OR "father attach*" OR "father infant attach*" OR "father daughter attach*" OR "father son attach*" OR "father to infant attach*" OR "father to daughter attach*" OR "father to son attach*" OR "paternal infant attach*" OR "paternal daughter attach*" OR "paternal son attach*" OR "paternal to infant attach*" OR "paternal to daughter attach*" OR "paternal to son attach*" OR "paternal fetus attach*" OR "father fetus attach*" OR "paternal foetus attach*" OR "father foetus attach*" OR "paternal to fetus attach*" OR "father to fetus attach*" OR "paternal to foetus attach*" OR "father to foetus attach*" OR "paternal child attach*" OR "father child attach*" OR "paternal to child attach*" OR "father to child attach*" OR "paternal adolescent bond*" OR

"father adolescent bond*" OR "paternal to adolescent bond*" OR "father to adolescent bond*" OR "paternal adolescent attach*" OR "father adolescent attach*" OR "paternal to adolescent attach*" OR "father to adolescent attach*") OR (TI("bonding" OR "bond" OR "bonds" OR "bonded" OR bond*) AND TI(father* OR paternal*)) OR (TI("attachment" OR "attach*") AND TI(father* OR paternal*)) OR (TX("bonding" OR "bond" OR "bonds" OR "bonded" OR bond* OR attach*) AND TX("infant*" OR "child*" OR "daughter*" OR "son" OR "sons" OR "fetus*" OR "foetus*" OR "adolescent*") AND TI(father* OR paternal*)) OR (TI("bonding" OR "bond" OR "bonds" OR "bonded" OR bond* OR attach*) AND TX("infant*" OR "child*" OR "daughter*" OR "son" OR "sons" OR "fetus*" OR "foetus*" OR "adolescent*") AND TX(father* OR paternal*)) OR TX(("bonding" OR "bond" OR "bonds" OR "bonded" OR bond*) NEAR/2 (father* OR paternal*)))
